# Scientific Items

**Published:** 1887-01-01

**Authors:** 


### SCIENTIFIC ITEMS.

**Rare Metals.**—The necessity for minute accuracy in chemical analysis has just been illustrated in an important discovery by Dr. Strohecker, of Frankfort. Somewhat extensive diluvial deposits of brick clay exist at Hainstadt, near Seeligenstadt. The bricks made from this clay vary considerably in color, according to the temperature at which they are burnt, but the cause of the variation has never before been suspected. It now appears that the layers of this clay are singularly rich in several metals hitherto very scarce, particularly cerium, glucinum, lanthanum, didymium and yttrium. The first two of these metals are present in such quantities that a more abundant supply may be expected. Ceria, in the form of hydrate, constituted 9.4 and 13.4 per cent. of the clay in two layers analyzed, and the color of the bricks seem to be mainly determined by its presence, for the quantity of iron present was very small. The discovery is therefore of immediate value, and wi l doubtless lead to further researches on the elements, which may prove to have much more importance in the economy of nature than has been supposed. It is evident that we must not neglect these little known elements, for, apart from their scientific interest, we cannot tell what undiscovered uses may lie in them. We do not know, indeed, whether they are really as scarce as has been supposed.—*Scientific American.*

**Aerial Navigation.**—The power of flying, being denied to man, has always been one of the objects most desired by him, though hitherto he has not succeeded in attaining it. If there were any large birds feeding on grains and possessing strong flying powers, they would no doubt have been domesticated long since, and made subservient to man's use, like horses and other animals. But, unfortunately, all large birds possessing strong wing power are carniverous and untamable, so we shall have to rest content with terrestrial locomotion till we have succeeded in solving the mechanical problem of propelling and steering balloons. We are still a long distance from this result, and it is at least very doubtful whether it will ever be attained.—*Ibid.*

**Another Electric Motor.**—A Third Avenue elevated car, brilliantly lighted with Edison incandescent lamps, recently made trips on the Thirty-fourth street branch of the elevated railroad in this city. The car was filled with a crowd ot interested electricians, for the Sprague electric motor was on trial. Notwithstanding the unfavorable condition of rain and a rusty track, the test was a successful one, and the fact that the car was both lighted, heated and propelled by electricity, and that the station platforms were similarly illuminated, seems to show that comfort and rapid transit are both to be increased by the use of electricity.

The Sprague motor is carried on the truck of the regular car, and differs from all other systems in the fact that all its parts and movements are controllable by electricity. On this trial the speed of the car was made to vary from twenty-three miles an hour to a bare crawl. It stopped, switched and reversed satisfactorily. No brake was used, the car being stopped by electricity. Stopping turns the motor into a generator, thereby saving much of the loss of electricity which happens in other systems. The electricity was supplied by two wires from a house half a mile away. Three tracks were employed, one wire being attached to the two outside tracks and the other to the middle track. The potential used was 600 volts. Mr. F. J. Sprague is the inventor of the new motor. His machine weighs only a ton, while the steam locomotives now in use weigh twenty tons. The motor is attached to each car, thus distributing the weight.—*Ibid.*

**Late Discoveries.**—The medical journals for the last ten years have given accounts of wonderful discoveries in surgical science and of their application in practice — the filling up of large, deep wounds with sponge, and the organization and assimilation of the latter; skin grafting, bone grafting, and the successful adjustment and regrowth of fingers. Recently two other wonderful discoveries have been reported. One is the organization of rubber within the animal tissues; the other, the organizing of blood-clots, their formation into new tissue, and the application of them to the surer and better healing of surgical wounds.

As to the first, it appears that Prof. Vanlair, of France, had, in a certain case, inserted a drainage tube, of ordinary gray vulcanized rubber, one and one-fourth inches in length and one-fifth in diameter, and that this, at the end of seven months, seemed to have undergone partial absorption.

But on examining it with a microscope, it was found that the substance of the rubber had become truly organized; that the lower end of the tube had become fully assimilated to the surrounding tissue, and had wholly lost its original form; that the part of the tube next above this had lost its original shapeless appearance and had acquired a complex structure, showing fine connecting tissue fibres, with cells of various forms between them, and very numerous capillary blood vessels.

The other discovery was by Schede, a German expert. The Boston *Med. and Surg. Journal* says: "His reported results are almost marvelous; the blood fills the wound-cavity completely, clots and is gradually replaced by permanent tissue formation. By this method resection (amputation) of large joints has healed by primary union, and large portions of the articular ends of bone have been removed without impairment of their articular function. Two hundred and forty-one operations are recorded by Schede, nearly all of which have healed under one dressing by primary union."

These operations included the amputation of forty large joints, with thirty-seven recovering, with no change of dressing and no leakage. The wound having been duly prepared, the blood is let in and left to organize, the whole being covered with protective silk and other dressing.—*Ex.*

